# Laparoscopic Distal Pancreatectomy Using Three-Dimensional Computer Graphics for Surgical Navigation With a Deep Learning Algorithm: A Case Report

**DOI:** 10.7759/cureus.55907

**Published:** 2024-03-10

**Authors:** Ryoichi Miyamoto, Masahiro Shiihara, Mitsugi Shimoda, Shuji Suzuki

**Affiliations:** 1 Department of Gastroenterological Surgery, Ibaraki Medical Center, Tokyo Medical University, Ibaraki, JPN

**Keywords:** ai, 3dcg, deep learning algorithm, pancreatic surgery, laparoscopic distal pancreatectomy

## Abstract

We have demonstrated the utility of SYNAPSE VINCENT^®^ (version 6.6; Fujifilm Medical Co., Ltd., Tokyo, Japan), a 3D image analysis system, in semi-automated simulations of the peripancreatic vessels, pancreatic ducts, pancreatic parenchyma, and peripancreatic organs using an artificial intelligence (AI) engine developed with deep learning algorithms. Furthermore, we investigated the usefulness of this AI engine for patients with pancreatic cancer. Here, we present a case of laparoscopic distal pancreatectomy with an extended surgical procedure performed using surgical simulation and navigation via an AI engine.

An 80-year-old woman presented with abdominal pain. Enhanced abdominal computed tomography (CT) revealed main pancreatic duct dilatation with a maximum diameter of 40 mm. Furthermore, there was a 17 mm cystic lesion between the pancreatic head and the pancreatic body and a 14 mm mural nodule in the pancreatic tail. Thus, the lesion was preoperatively diagnosed as an intraductal papillary carcinoma (IPMC) of the pancreatic tail and classified as T1N0M0 stage IA according to the 8th edition of the Union for International Cancer Control guidelines. The present patient had laparoscopic distal pancreatectomy and regional lymphadenectomy. In particular, since it was necessary to include the cystic lesion in the pancreatic neck, pancreatic resection was performed at the right edge of the portal vein, which is closer to the head of the pancreas than usual. We routinely employed three-dimensional computer graphics (3DCG) surgical simulation and navigation, which allowed us to recognize the surgical anatomy, including the location of pancreatic resection. In addition to displaying the detailed 3DCG of the surgical anatomy, this technology allowed surgical staff to share the situation, and it has been reported that this approach improves the safety of surgery. Furthermore, the remnant pancreatic volume (47.6%), pancreatic resection surface area (161 mm^2^), and thickness of the pancreatic parenchyma (12 mm) at the resection location were investigated using 3DCG imaging. Intraoperative frozen biopsy confirmed that the resection margin was negative. Histologically, an intraductal papillary mucinous neoplasm with low-grade dysplasia was observed in the pancreatic tail. No malignant findings, including those related to the resection margin, were observed in the specimen. At the 12-month postoperative follow-up examination, the patient’s condition was unremarkable.

We conclude that the SYNAPSE VINCENT^®^ AI engine is a useful surgical support for the extraction of the surrounding vessels, surrounding organs, and pancreatic parenchyma including the location of the pancreatic resection even in the case of extended surgical procedures.

## Introduction

Pancreatic surgery requires accurate recognition of anatomical variations and their relationships regarding the tumor location to peripancreatic vessels or organs in order to determine the location of the pancreatic resection. In particular, pancreaticoduodenectomy or distal pancreatectomy is usually required to confirm the accurate recognition of the pancreaticoduodenal arcade. This arcade, which includes the gastroduodenal artery (GDA) and the inferior pancreaticoduodenal artery, often has anatomical variation [1,2]. To avoid or minimize the risk of surgical complications, it is also essential to be aware of the blood supply of the hepatic artery and the anatomy of the vulnerable veins, such as the left gastric vein, the inferior mesenteric vein, and the jejunal vein [3-5].

Recent advances in radiological diagnostic imaging workstations have facilitated the creation of three-dimensional computer graphics (3DCG) with vascular and organ images. In 2013, we continued to develop and extend our 3DCG simulation technique, which is already being used for liver and pancreatic surgery [6,7]. Previously, we have demonstrated that 3DCG simulation of pancreatic surgery contributed to a better understanding of surgical anatomy by surgical staff, improved short-term outcomes, and predicted the precise position of the main pancreatic duct in the pancreatectomy planes and residual pancreatic volume [8-10].

In 2022, we previously reported that the usefulness of SYNAPSE VINCENT® (version 6.6; Fujifilm Medical Co., Ltd., Tokyo, Japan) in a semi-automated simulation technique of pancreatic surgery using an artificial intelligence (AI) engine developed with deep learning algorithms [11].

Here, we present a case of laparoscopic distal pancreatectomy with an extended surgical procedure performed using surgical simulation and navigation using an artificial intelligence (AI) engine.

## Case presentation

An 80-year-old woman presented with abdominal pain. The patient had no significant past medical history, and her general physical examination was normal. There were no abnormal laboratory values, including those suggestive of pancreatitis.

**Figure 1:**
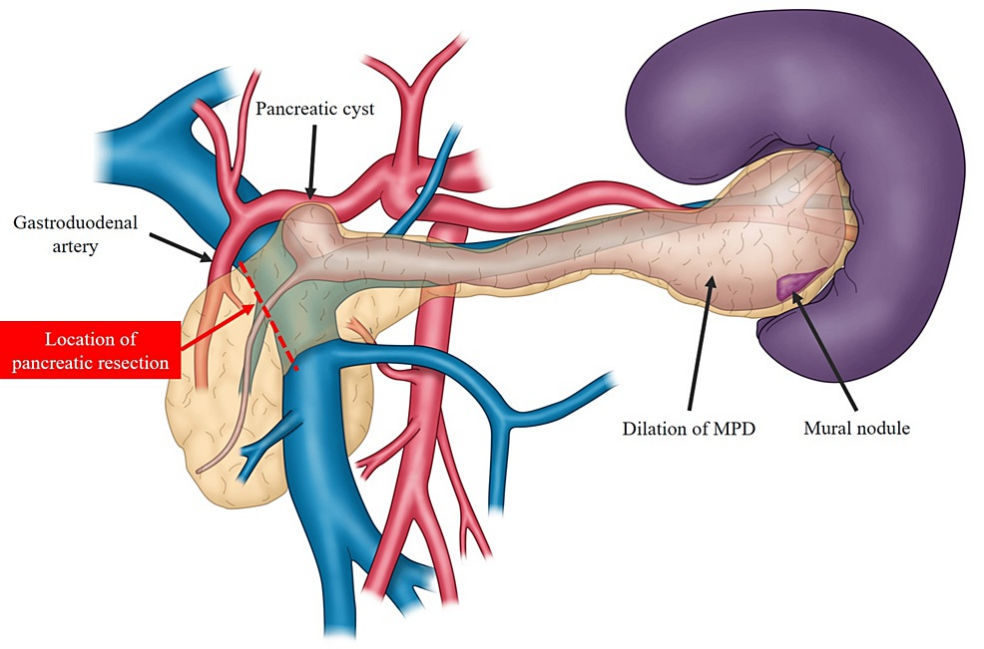
Schema showing the anatomical structures in the present case. This schema was originally created by the author based on preoperative images. A 17 mm cystic lesion in the pancreatic neck and a 17 mm mural nodule in the pancreatic tail with MPD dilatation and a maximum diameter of 40 mm were observed. The location of pancreatic resection was assumed to be the right edge of the portal vein. Abbreviations: MPD, main pancreatic duct.

Computed tomography (CT) of the abdomen revealed main pancreatic duct dilatation with a maximum diameter of 40 mm. Furthermore, there was a 17 mm cystic lesion between the pancreatic head and the pancreatic body and a 14 mm mural nodule in the pancreatic tail (Figure 1).

Furthermore, the remnant pancreatic volume (47.6%), pancreatic resection surface area (161 mm^2^), and thickness of the pancreatic parenchyma (12 mm) at the resection location were investigated using 3DCG images.

**Figure 2:**
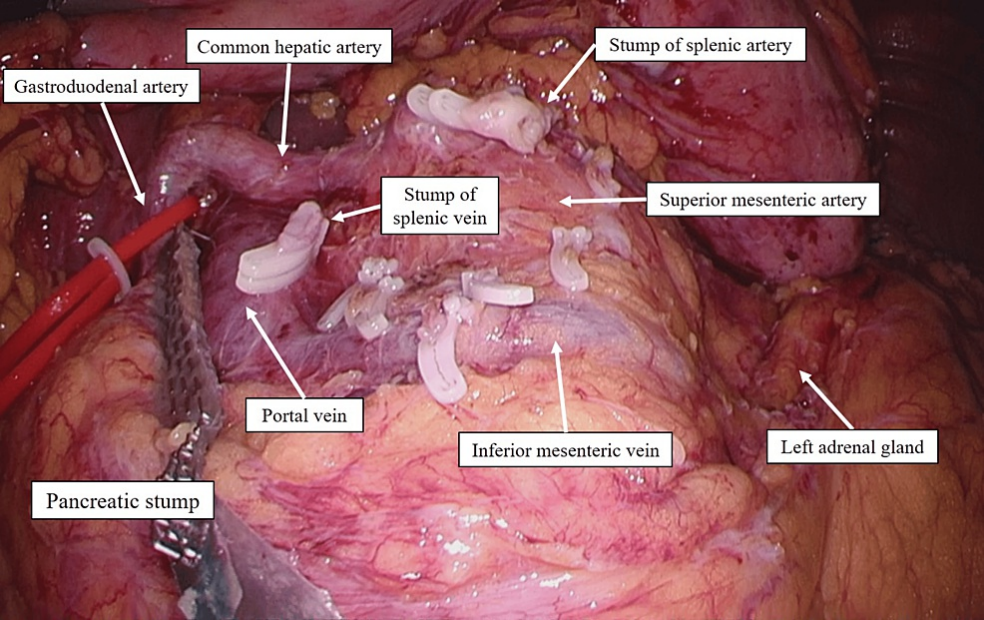
Intraoperative findings after pancreatic resection. Pancreatic resection was performed with a linear stapler at the right edge of the portal vein.

Pancreatic resection was performed using a linear stapler at the right edge of the portal vein (Figure 2). Intraoperative frozen biopsy confirmed that the resection margin was negative. At the 12-month postoperative follow-up examination, the patient’s condition was unremarkable.

Therefore, the lesion was diagnosed as an IPMC of the pancreatic tail and classified as T1N0M0 stage IA according to the 8th edition of the Union for International Cancer Control (UICC) guidelines.

**Figure 3:**
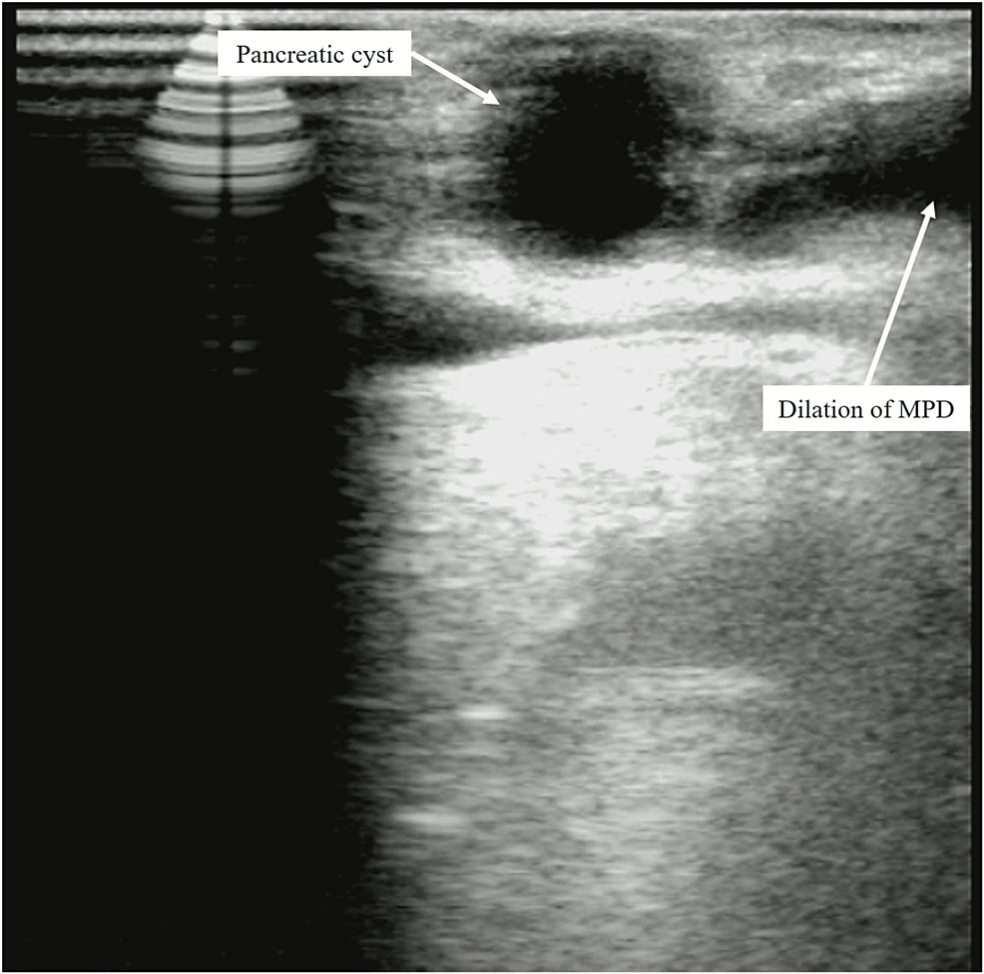
IOUS revealed a 17 mm cystic lesion in the pancreatic neck with MPD dilation. Abbreviations: IOUS, intraoperative ultrasound; MPD, main pancreatic duct.

**Figure 4:**
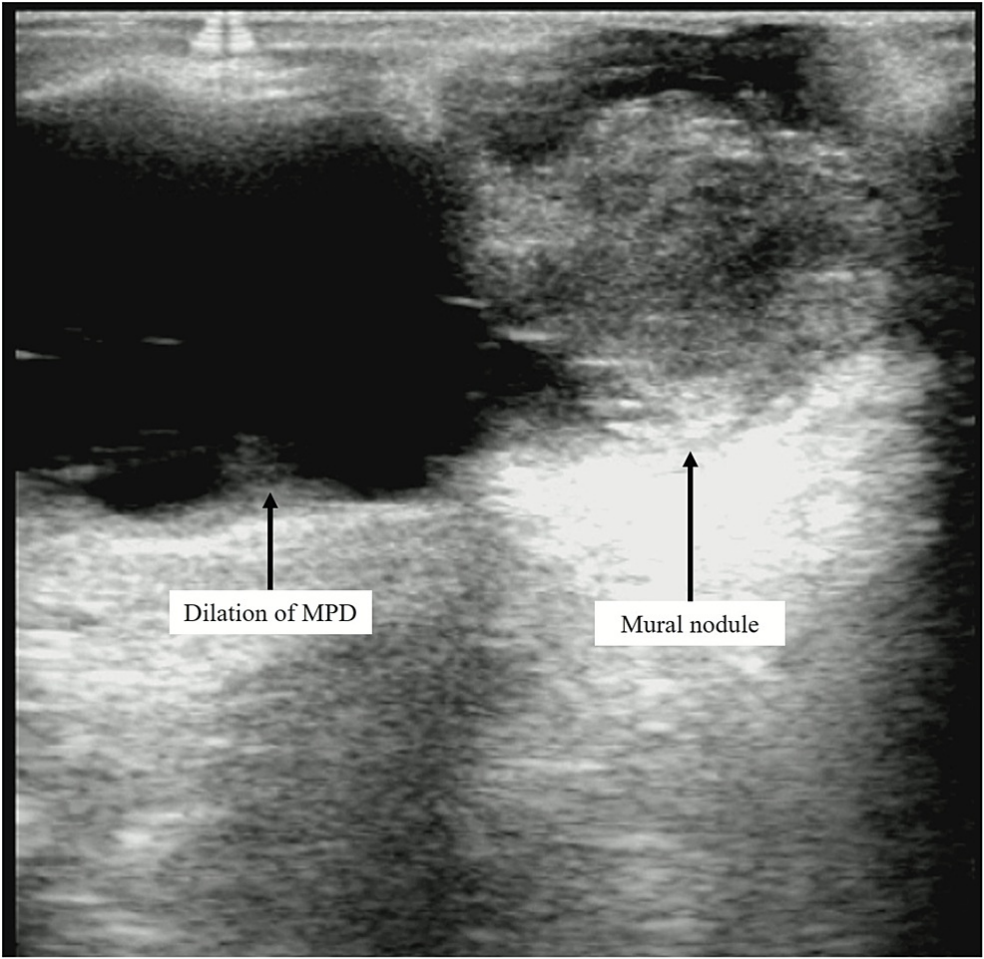
IOUS revealed a 14 mm mural nodule in the pancreatic tail with MPD dilation. Abbreviations: IOUS, intraoperative ultrasound; MPD, main pancreatic duct.

The present patient had laparoscopic distal pancreatectomy and regional lymphadenectomy. In particular, since it was necessary to include the cystic lesion in the neck, pancreatic resection was performed at the right edge of the portal vein, which is closer to the head of the pancreas than usual (Figure 1). Intraoperative ultrasound (IOUS) revealed a 17 mm cystic lesion in the pancreatic neck and a 14 mm mural nodule in the pancreatic tail (Figures 3-4).

**Figure 5:**
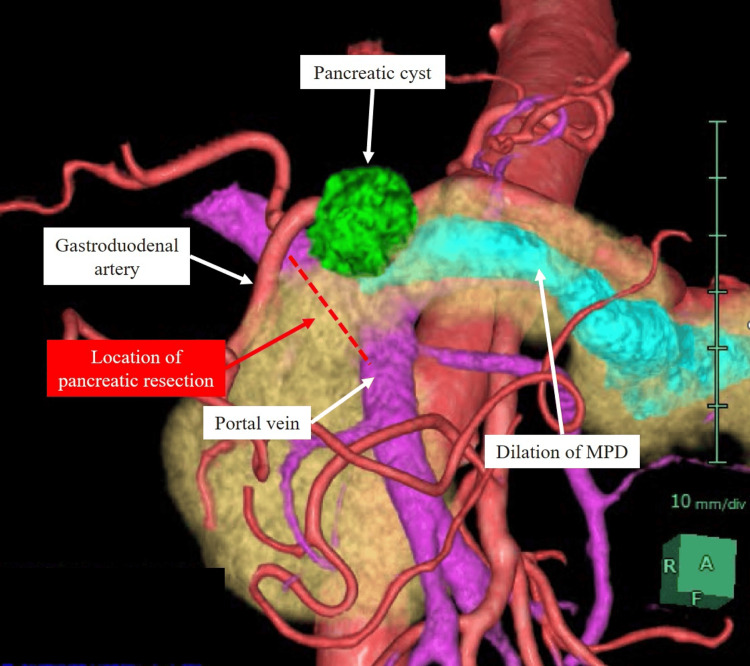
3DCG image from the present patient. This view is an anterior 3DCG image. The arteries were symbolized by the red color; the veins the purple; the pancreas the yellow; the pancreatic duct the blue; the pancreatic cyst the green. Abbreviations: 3DCG, three-dimensional computer graphics.

**Figure 6:**
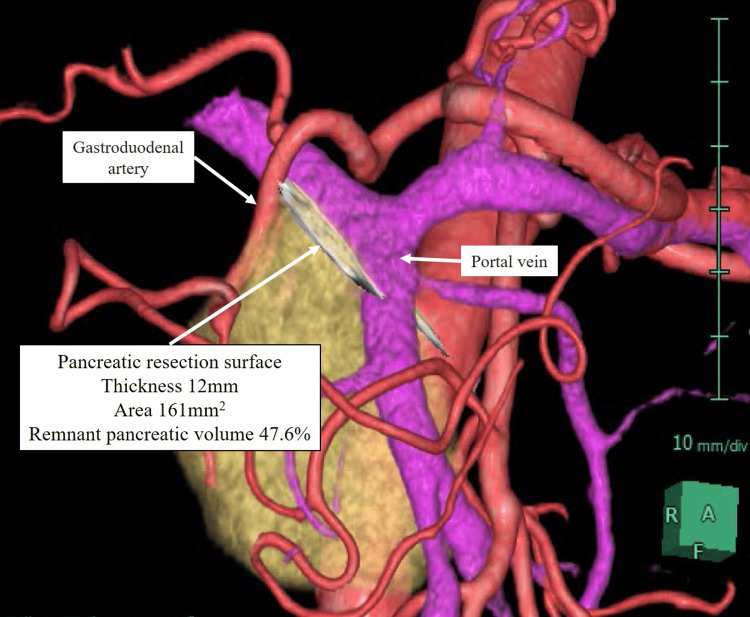
3DCG image assuming resection of the pancreas at the right end of the portal vein. The arteries were symbolized by the red color; the veins the purple; the pancreas the yellow. Abbreviations: 3DCG, three-dimensional computer graphics.

We routinely employed 3DCG surgical simulation and navigation, which allowed us to recognize the surgical anatomy, including the location of the pancreatic resection (Figures 5-6). In addition to displaying the detailed 3DCG surgical anatomy, this technology allows surgical staff to share the situation, and it has already been reported that it increases the safety of surgery [8-10].

Furthermore, the remnant pancreatic volume (47.6 %), pancreatic resection surface area (161 mm2), and thickness of the pancreatic parenchyma (12 mm) at the resection location were investigated using 3DCG images.

Pancreatic resection was performed using a linear stapler at the right edge of the portal vein (Figure 6). Intraoperative frozen biopsy confirmed that the resection margin was negative. At the 12-month postoperative follow-up examination, the patient’s condition was unremarkable.

**Figure 7:**
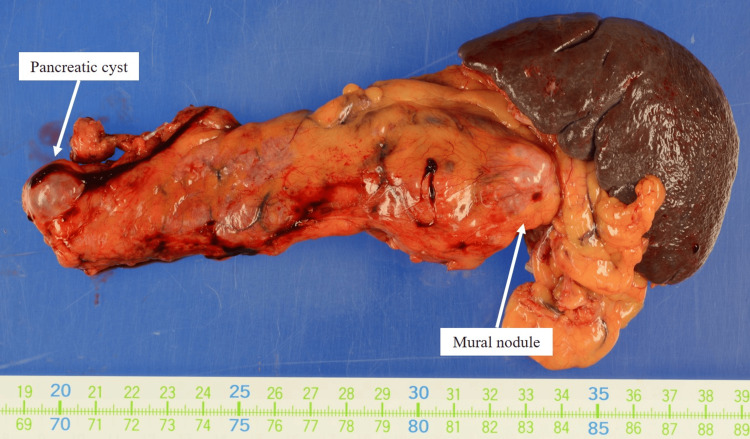
Histopathological findings in the present case. An intraductal papillary mucinous neoplasm with low-grade dysplasia was observed in the pancreatic tail. No malignant findings were observed in the specimen, including at the resection margin.

Histologically, an intraductal papillary mucinous neoplasm with low-grade dysplasia was observed in the pancreatic tail. No malignant findings, including those relating to the resection margin, were observed in the specimen (Figure 7).

## Discussion

We successfully performed a laparoscopic distal pancreatectomy with an extended surgical procedure using surgical simulation and navigation with this AI engine. By displaying 3DCG images in real-time during surgery and using them for intraoperative simulation or navigation, it was useful in understanding the surgical anatomy, including the location of pancreatic resection, not only for normal surgical procedures but also for particularly extended surgical procedures.

In this case, we found that it was necessary to perform the pancreatic resection closer to the head of the pancreas than usual, so it was important to evaluate the surrounding blood vessels, especially the course of the GDA, the location of the pancreatic resection and the use of 3DCG images.

Previously, we demonstrated that the newly developed AI engine with deep learning algorithms helped to accurately extract the pancreatic parenchyma and peripancreatic vessels. Furthermore, we reported that even in pancreatic cancer patients, there was no change in the extraction accuracy of the AI engine in terms of tumor stage, size, or location [11].

Regarding the usefulness of 3DCG images created by AI engines, although confirmation of the 3DCG image by the surgeon is necessary, the image extraction can be performed semi-automatically without the labor cost of conventional software, and can be widely used in pancreatectomy. Furthermore, pancreatectomy can be performed using 3DCG images visualizing the anatomical structures around the pancreas extracted by the AI engine. The pancreas can be isolated at any position, and the anatomical relationship between the isolated pancreas and surrounding blood vessels and organs, the predicted position of the pancreatic duct relative to the isolated pancreas, the automatically resected and remaining pancreatic regions, the shape and surface area of the resected pancreas surface, and the thickness of the resected pancreas can also be automatically obtained from 3DCG images. The 3DCG image can also be used to automatically determine the shape, surface area, and thickness of the resected pancreas.

Another clinical benefit of preoperative 3DCG imaging is assumed to be the assessment of residual pancreatic volume, pancreatectomy surface area, and the thickness of the pancreatic parenchyma at the resection site. Our previous studies have reported that postoperative pancreatic volume is closely related to postoperative pancreatic endocrine insufficiency and is useful in predicting long-term postoperative prognosis in pancreatic cancer patients [12,13].

With respect to the thickness of the pancreatic parenchyma at the resection site, it has been previously reported that the appropriate combination of stapler cartridge size and thickness is one of the important considerations during staple closure [14,15]; Kawai et al. previously reported that a pancreatic thickness greater than 12 mm increases the closure and reported a significantly increased risk of pancreatic fistula formation after distal pancreatectomy [16]. Low staple height can cause laceration and avulsion of the hard or thick pancreatic parenchyma. Conversely, high staple height may result in inadequate sealing of the pancreatic parenchyma, including the main pancreatic duct, causing leakage and bleeding from the pancreatic stump. Therefore, accurate preoperative assessment of pancreatic thickness contributes to appropriate pancreatic stump closure. In this patient, the thickness of the pancreas at the site of pancreatic resection was 12 mm, which could be measured by 3DCG images preoperatively, making it possible to select an appropriate staple size.

## Conclusions

We conclude that the SYNAPSE VINCENT® AI engine as a surgical support technique can be useful even in case of the extended surgical procedures. Preoperative consideration of various parameters including residual pancreatic volume, pancreatectomy surface area, and the thickness of the pancreatic parenchyma at the resection site, which could be easily calculated using 3DCG, would contribute to safer pancreatectomy.
